# Detection of *Toxoplasma gondii* DNA by qPCR in the feces of a cat that recently ingested infected prey does not necessarily imply oocyst shedding

**DOI:** 10.1051/parasite/2016029

**Published:** 2016-07-22

**Authors:** Marie-Lazarine Poulle, Marie-Amélie Forin-Wiart, Émilie Josse-Dupuis, Isabelle Villena, Dominique Aubert

**Affiliations:** 1 Université de Reims Champagne-Ardenne, SFR Cap-Santé, EA3800-PROTAL, UFR de Médecine 51 rue Cognacq-Jay 51095 Reims Cedex France; 2 Université de Reims Champagne-Ardenne, CERFE 5 rue de la Héronnière 08240 Boult-aux-Bois France

**Keywords:** *Toxoplasma gondii*, Domestic cat, Oocyst, Coprodiagnosis, qPCR

## Abstract

Detection of *Toxoplasma gondii* DNA in cat feces is considered indicative of the presence of *T. gondii* oocysts. This study aims to demonstrate that the high sensitivity of qPCR can lead to *T. gondii* DNA detection in cat feces in the absence of oocysts. A cat immune to toxoplasmosis was fed with a mouse experimentally infected with *T. gondii.* Detection of DNA of this parasite was performed by qPCR on feces passed: (i) on the day the cat ingested the infected prey; (ii) during the three previous days; and (iii) during the three following days. The kinetics of qPCR results are clearly not linked to oocyst shedding and this result demonstrates that qPCR can detect *T. gondii* DNA related to bradyzoites from an infected prey, in the absence of oocysts. Caution is thus recommended when interpreting *T. gondii* qPCR results for samples of cat feces.

## Abstract (fr)

La détection d’ADN de *Toxoplasma gondii* dans les fèces de chats est considérée comme révélatrice de la présence d’oocystes. Cette étude a pour objectif de démontrer que la grande sensibilité de la qPCR peut conduire à la détection de l’ADN de *T. gondii* dans les fèces de chat en l’absence d’oocystes. Un chat immunisé contre la toxoplasmose a été nourri avec une souris expérimentalement infectée par *T. gondii.* La détection d’ADN de ce parasite a été réalisée par qPCR sur les fèces émises : (i) le jour où le chat a ingéré la proie infectée ; (ii) durant les trois jours précédents ; (iii) durant les trois jours suivants. La cinétique des résultats de qPCR n’est clairement pas celle d’une émission d’oocystes et ce résultat démontre que la qPCR peut détecter l’ADN de *T. gondii* lié aux bradyzoïtes d’une proie infectée, en l’absence d’oocystes. La prudence est donc recommandée pour l’interprétation des résultats de qPCR de *T. gondii* sur fèces de chats.

## Introduction

*Toxoplasma gondii* is a widespread zoonotic protozoan parasite that infects all warm-blooded vertebrates, including bird and mammal species. It has three infectious stages: tachyzoites (rapidly multiplying cells, circulating form), bradyzoites (tissue cyst form), and sporozoites (in sporulated oocysts). *Toxoplasma gondii* has both a simple and a complex life cycle, in which felids are the definitive hosts, and all warm-blooded vertebrate species – including humans – can serve as intermediate hosts [3]. Felids are the only animals in which the parasites can undergo sexual replication. Consequently, felids serve as the main reservoir of infection for other animals and humans [4]. Sexual replication generates unsporulated oocysts that are released into the intestinal lumen and pass into the environment along with feces. Oocyst shedding generally starts three days at the earliest after definitive host infection and lasts no more than 21 days, but may recur in the case of immunosuppression [2–4]. Sporogony occurs outside the host and leads to the development of infectious oocysts, which remain viable in the environment for months to years [10]. Among felids, the domestic cat, *Felis silvestris catus*, is the species most often associated with a risk of human infection [6].

**Table 1.** Results of qPCR detection of *Toxoplasma gondii* DNA extracted from the feces of a housebound cat that had antibodies against this parasite and that had ingested a *Toxoplasma gondii*-infected prey on day 4. One stool analyzed per day. The run numbers are given for each of the three samples analyzed per stool. NA: no *T. gondii* DNA detected in the sample.

Days before and after ingestion of a *T. gondii*-infected prey	qPCR result	Run numbers (Ct)
Day 1	−	NA/NA/NA
Day 2	−	NA/NA/NA
Day 3	−	NA/NA/NA
Day 4: ingestion of an infected prey	−	NA/NA/NA
Day 5	+	35.04/35.19/35.75
Day 6	+	NA/NA/41.20
Day 7	+	NA/NA/39.97
Day 8	−	NA/NA/NA

Accurate detection of oocysts in cat feces is of special concern to prevent toxoplasmosis because the risk of human infection and environmental contamination is closely linked with cats that actively shed oocysts (review in [5]). Oocyst detection in cat feces has been carried out by microscopy for decades, but the sensitivity of this method is low [15]. Conversely, the mouse bioassay is sensitive [17], but it is costly and time-consuming. A molecular approach for the coprodiagnosis of *T. gondii*, as sensitive and as specific as the bioassay, was developed by Salant et al. [14]. This copro-PCR can detect infective oocysts during cat infection [15]. However, Mancianti et al. [12] suggested that the significance of *T. gondii* copro-PCR positivity should be carefully evaluated because this finding could be related not only to oocysts but also to the presence of DNA from asexual stages of *T. gondii*. The risk of detecting *T. gondii* DNA unrelated to oocysts is even higher with qPCR because of the high sensitivity of this method.

Such qPCR that targets repetitive DNA sequences for detecting and quantifying oocysts in biological samples has gained popularity in recent years [8, 16]. As compared to conventional PCR, qPCR has better sensitivity and a better ability to overcome contamination and reduce reaction times [1]. Furthermore, it allows for highly sensitive quantification of parasitic DNA in carnivore feces [7]. Low levels of *T. gondii* genomes have been detected by qPCR in the feces of cats 5 days after they were infected with a low dose of 10 bradyzoites [2]. Based on this result, the authors supposed that qPCR may detect fragments of *T. gondii* DNA in the absence of oocysts, but no data were available to test this assumption until the present study.

This study aims to demonstrate that *T. gondii* qPCR positivity in cat feces may actually be associated with the detection of DNA from bradyzoites from an infected prey, in the absence of oocysts.

## Materials and methods

### *Toxoplasma gondii* infection in a cat

The animal experiments were performed in 2012 according to French law concerning ethics and laboratory animals; the animals were bred in the laboratory animal facility of the CHU of Reims (Approval No. B 51-454, dated 2008). The experiment was conducted in November 2012 on a 31-month-old, intact, housebound, male domestic cat. This usually free-roaming cat originating from a rural area had to be confined for 31 days to treat a wound. Serum had been previously drawn under anesthesia in March 2011, August 2011, and March 2012 – i.e. 20 months, 15 months, and 8 months before the experiment, to be screened for *T. gondii*-specific antibodies with a Modified Agglutination Test (MAT) positive at a 1:25 dilution. The results of the three MAT assays showed the presence of past infection through the detection of *T. gondii-*specific antibodies (IgG). Sera were also systematically tested for feline immunodeficiency virus (FIV) and Feline leukemia virus (FeLV), and results indicated that the cat was free of these infections.

The experiment was initiated once the cat had completely recovered from his wound and lasted 8 days. Commercial dry pet food and tap water were available *ad libitum*. From days 1 to 8, all feces were collected daily from a litter tray, sieved out, placed in plastic bags, labeled, and stored at −20 °C. The litter box was then cleaned. On day 4, the cat was fed with an outbred female Swiss Webster mouse (Charles River Laboratories, France) infected with the genotype II *T. gondii* ME49 strain. The cat ate the whole infected mouse in one meal. DNA extraction and *T. gondii* DNA detection by qPCR were performed on each feces sample emitted between day 1 and day 8.

### DNA extraction

Total DNA was extracted from approximately 500 mg of fecal material taken from all scat parts of the feces. All the extractions were performed by Spygen (SAS SPYGEN, France) using the Qiagen DNA Stool Mini kit and following the manufacturer’s protocol. Extractions were performed in a room dedicated to degraded or rare DNA. Extraction blanks (containing no DNA) were included as negative controls to test for contamination. The success of the DNA extraction was assessed by qPCR amplification with a species-specific primer. The extracts were stored at −20 °C until qPCR amplification.

### Real-time PCR conditions

DNA extracts were subjected to real-time quantitative PCR (qPCR), targeting a specific 529 bp DNA repeat element [13]. Quantitative PCR was performed on an iQ5 instrument (BIORAD), as follows. A *T. gondii*-specific target gene (AF 487550) was detected and amplified with a labeled Taqman probe (6FAM-ACG CTT TCC TCG TGG TGA TGG CG-TAMRA) and DNA oligonucleotide primers (5′-AGA GAC ACC GGA ATG CGA TCT-3′ and 5′-CCC TCT TCT CCA CTC TTC AAT TCT-3′) synthesized by Eurogentec S.A., Seraing, Belgium (as used by [9]). The amplification mixture consisted of 12.5 μL of 2× reaction mixture (Platinum Quantitative PCR Supermix UDG, Invitrogen), 4 mM MgCl_2_, 0.5 μM of each oligonucleotide primer, 0.2 μM *Taq*man probe, 1 μL of 1% bovine serum albumin (BSA) for each sample before amplification to prevent PCR inhibition, and 5 μL of template DNA in a final volume of 25 μL. The reaction mixture was initially incubated for 3 min at 50 °C to allow for uracil-N-glycosylase (UNG) enzymatic activity. This incubation was followed by a second incubation of 3.3 min at 95 °C to denature the DNA template, to inactivate the UNG enzyme, and to activate the Platinum *Taq* DNA Polymerase. Samples were amplified as follows: 45 cycles of denaturation at 95 °C for 15 s and annealing/extension at 60 °C for 1 min. Each sample was split and tested in triplicate. Negative controls were included from DNA extraction to PCR amplification, and each PCR plate contained two controls: a negative and a positive control. Negative results were systematically obtained for the negative control. Results were expressed as the number of cycles required to reach the detection threshold (Ct). Laboratory analyses were performed at the Laboratoire de Parasitologie-Mycologie, EA3800, in Reims, France.

## Results and discussion

From day 1 to day 8, the cat passed one scat per day. *Toxoplasma gondii* DNA was not detected in feces before ingestion of the infected mouse, including the day the mouse was ingested (day 1–4, Table 1). Subsequently, *T. gondii* DNA was detected in all three replicates performed on the day 5 sample (*n* + 1 day after ingesting the infected prey) and in at least one of the triplicates performed on the day 6 and day 7 samples (Table 1). All three qPCR replicates performed on day 8 were negative (Table 1).

Although based on a single case, the kinetics of these results clearly demonstrate that qPCR can detect *T. gondii* DNA from bradyzoites in the absence of oocyst shedding:

- The qPCR negativity between day 1 and day 4 confirms that the cat, which had experienced a past infection with *T. gondii* and was thus considered as an immune individual, was not in an oocyst excretion or re-excretion period before it ingested the infected prey.
- If the ingestion of bradyzoites *via* the infected prey had induced a re-shedding of oocysts, qPCR positivity of the feces would have started three days at the earliest after re-infection (i.e. day 7 in our experiment) and would have persisted on day 8 (for a review of the kinetics of *T. gondii* infection and re-infection see [3]). We did not record such a pattern.
- The peak of qPCR positivity was noted as early as one day after prey ingestion (day 5) and was followed by a rapid decrease in the DNA amounts in the next two days (day 6 and day 7) to disappear on the third day (day 8). This kinetic pattern clearly supports the assumption that *T. gondii* DNA detected from days 5 to 7 originated from the infected prey.

A microscopic examination of stool samples after flotation would have confirmed the absence of oocysts but this was not necessary to conclude that the ingestion of infected prey can result in *T. gondii* qPCR positivity in the feces of a cat that does not excrete oocysts. This might have consequences in the case of challenge studies to evaluate the efficacy of vaccines [2] or in field surveys of *T. gondii* infection in free-roaming cats on the basis of feces samples [11, 12], since only feces containing oocysts are of epidemiological relevance. Caution is thus recommended when interpreting *T. gondii* qPCR results for cat feces samples.
